# Running on an unpredictable irregular surface changes lower limb biomechanics and subjective perception compared to running on a regular surface

**DOI:** 10.1186/1757-1146-7-S1-A80

**Published:** 2014-04-08

**Authors:** Thorsten Sterzing, Charlotte Apps, Rui Ding, Jason Tak-Man Cheung

**Affiliations:** 1 Sports Science Research Center, Li Ning (China) Sports Goods Co Ltd, Beijing 101111, China; 2 School of Sport and Exercise Sciences, Liverpool John Moores University, Liverpool, L3 3AF, UK

## Background

Irregular surface conditions, for instance, are present during trail running. Modified treadmills can be used to produce such surface conditions in a laboratory environment [1]. Gait variability on uneven shoe-surface interfaces is increased in walking [2,3], hence the same may apply to running. This study examined the effects of an unpredictable irregular surface (UIS) on lower limb biomechanics, locomotion variability, and subjective perception during treadmill running.

## Methods

Seventeen young, male, active participants ran at 8 km/h on a treadmill with predictable regular surface (PRS) and with UIS. The UIS was created by randomly attaching EVA dome shaped inserts (ط: 140 mm) of different height (10 mm and 15 mm) and hardness (40 and 70 Asker C) to the treadmill. In-shoe plantar pressures (200 Hz, Pedar X System, Novel, Germany), lower limb kinematics (200 Hz, Vicon Peak, United Kingdom), and EMG signals of five lower limb muscles (3000 Hz, Telemyo 2400 G2, Noraxon, USA) were recorded. Eight perception items were assessed subjectively (9-point Likert Scale). Biomechanical parameter mean magnitudes and mean standard deviations, as variability measure, of 16 steps were calculated. Variables were compared between surfaces by Wilcoxon signed rank tests (p<.05).

## Results

Step length decreased while step frequency increased on UIS (p<.05). In-shoe pressure relative load magnitudes on UIS were increased at medial midfoot (p<.05), and decreased at lateral forefoot (p<.05). Relative load variability increased for all regions (p<.05). Runners had a flatter and less dorsiflexed foot strike (Table 1), alongside increased knee and hip flexion on UIS (p<.05). Whereas all sagittal joint angle magnitudes differed significantly, only knee and hip angles varied significantly more. Touchdown ankle inversion remained unchanged, whereas maximum eversion was significantly higher on UIS, and both were more variable (p<.05). Tibialis anterior and gastrocnemius medialis muscle activity magnitude and variability was similar, whereas peroneus longus activity was significantly increased, while not being more variable on UIS (Table 1). Subjectively, running on UIS was more challenging (p<.05).

**Table 1:** Magnitude (Mag) and variability (Var) of kinematic and EMG parameters, significant surface comparisons (PRS vs. UIS) indicated in bold.

	Sagittal plane angles [deg]				Normalized muscle activity during stance [%]					
	
	Shoe to Surface		Shoe to Shank		Tibialis Anterior		Gastrocnemius Med		Peroneus Longus	
	Mag	Var	Mag	Var	Mag	Var	Mag	Var	Mag	Var

**PRS**	20.8	2.1	9.7	1.3	24.4	3.3	42.1	5.6	42.7	5.8

**UIS**	17.0	2.7	7.1	1.9	22.8	3.5	43.5	5.4	46.8	7.7

**p-value**	**.001**	.102	**.001**	.055	.149	.492	.831	.586	**.025**	.068

## Conclusion

Runners consciously applied a more alert kinematic lower limb posture at touchdown on UIS, with lower limb position more consistent for distal sagittal joint angles. Similar muscular activity of tibialis anterior and gastrocnemius medialis indicates that general muscle activity applied was sufficient to compensate the perturbation level in this study regarding sagittal plane ankle motion. Running on UIS increased gait variability, thus stimulating enhancement of motor control patterns, resembling a positive training mechanism [4].
